# Retraction: Vitexin mitigates myocardial ischemia reperfusion-induced damage by inhibiting excessive autophagy to suppress apoptosis *via* the PI3K/Akt/mTOR signaling cascade

**DOI:** 10.1039/d1ra90024b

**Published:** 2021-01-21

**Authors:** Laura Fisher

**Affiliations:** Royal Society of Chemistry Thomas Graham House, Science Park, Milton Road Cambridge CB4 0WF UK advances-rsc@rsc.org

## Abstract

Retraction of ‘Vitexin mitigates myocardial ischemia reperfusion-induced damage by inhibiting excessive autophagy to suppress apoptosis *via* the PI3K/Akt/mTOR signaling cascade’ by Zhaobin Tang *et al.*, *RSC Adv.*, 2017, **7**, 56406–56416, DOI: 10.1039/C7RA12151B.

The Royal Society of Chemistry hereby wholly retracts this *RSC Advances* article due to concerns with the reliability of the data.

The images in the article were screened by an image integrity expert who confirmed that some of the western blot images in this paper had been duplicated in other articles. There are no common authors between the papers.

The control bands (GAPDH) presented in Fig. 2C and 4G of this paper are identical. In addition, these bands are identical to the western blot control bands (GAPDH) presented in Fig. 4A of ref. 1.

One of the blots in the control band (GAPDH) in Fig. 2C has also been reused as a blot in Fig. 3D of ref. 2.

The p62 band in Fig. 4A has been duplicated as the Col II band in Fig. 3B of ref. 2.

The authors were asked to provide the raw data for this article but did not respond. Given the significance of the concerns about the validity of the data, and the lack of raw data, the findings presented in this paper are not reliable.

The authors have been informed but have not responded to any correspondence regarding the retraction.

Signed: Laura Fisher, Executive Editor, *RSC Advances*

Date: 7^th^ January 2021

## Supplementary Material
